# Considerations when using the significance analysis of microarrays (SAM) algorithm

**DOI:** 10.1186/1471-2105-6-129

**Published:** 2005-05-29

**Authors:** Ola Larsson, Claes Wahlestedt, James A Timmons

**Affiliations:** 1 Center for Genomics and Bioinformatics, Karolinska Institutet, Berzelius Väg. 35. 171 77 Stockholm, Sweden

## Abstract

**Background:**

Users of microarray technology typically strive to use universally acceptable data analysis strategies to determine significant expression changes in their experiments. One of the most frequently utilised methods for gene expression data analysis is SAM (significance analysis of microarrays). The impact of selection thresholds, on the output from SAM, may critically alter the conclusion of a study, yet this consideration has not been systematically evaluated in any publication.

**Results:**

We have examined the effect of discrete data selection criteria (qualification criteria for inclusion) and response thresholds (out-put filtering) on the number of significant genes reported by SAM. The use of a reduced data set by applying arbitrary restrictions vis-à-vis abundance calls (e.g. from D-chip) or application of the fold change (FC) option within SAM (named the FC hurdle hereafter), can substantially alter the significant gene list when running SAM in Microsoft Excel. We determined that for a given *final *FC criteria (e.g. 1.5 fold change) the FC hurdle applied within Microsoft Excel SAM alters the number of reported genes above the *final *FC criteria. The reason is that the FC hurdle changes the composition of the control data set, such that a different significance level (q-value) is obtained for any given gene. This effect can be so large that it changes subsequent *post hoc *analysis interpretation, such as ontology overrepresentation analysis.

**Conclusion:**

Our results argue for caution when using SAM. All data sets analysed with SAM could be reanalysed taking into account the potential impact of the use of arbitrary thresholds to trim data sets before significance testing.

## Background

Response thresholds and exclusion criteria are applied when presenting a summary of a microarray study; otherwise the data set can be unmanageable. The precise effect of such criteria is, however, rarely discussed or investigated. We believe that many researchers are typically not aware of the effect that their chosen thresholds or 'qualification' criteria have had on their final set of "significant genes". A common example would be data exclusion. In studies using the Affymetrix platform the selection criteria typically uses the absent, moderate or present 'call' system, calculated with algorithms implemented in MAS5 (microarray suite 5 from Affymetrix) or D-chip [1]. The decision of whether the detection threshold is set to 10% or 20% (for example) will change the number of significant genes when the analysis is followed by the use of a parametric test with multiple testing corrections (for example). How predictable the imposition of such thresholds, on the composition of the output, is not clear.

A recent method to identify significant genes is "Significance Analysis of Microarrays" (SAM)[2]. An ISI search indicates that SAM is the most popular method employed for microarray analysis (635 citations of the original publication as of October 2004). In SAM, the relative difference (d(i)) is compared to the distribution of d(i) following random permutation of the sample categories. For each d(i), a certain proportion of all genes in the permutation set (control set) will be found to be 'significant' by chance and this parameter is then used to calculate a "False Discovery Rate" (FDR). This is presented as a q value for each gene in the final list of significant genes. The q-values are influenced by the variability in the data set. This implies that changes in the entire data set composition will affect the d(i) distribution in the permuted control set and thus the q-value assigned to a given gene. To what extent this alters the final output when using SAM or subsequent post-hoc analysis has so far not been discussed. As SAM is arguably the most widely utilized method in the microarray analysis field, we felt it was critical to evaluate these considerations.

### The number of reported significant genes is influenced by the FC setting within SAM and the use of "Present/Absent" calls for data inclusion

The first point to emphasise is that we are examining the effect of a variable FC setting, implemented in the Excel SAM, on a final gene list with a fixed 1.5 FC criteria. Our principle findings can be extended to any relevant final FC criteria but in the example we provide we have focused on a 1.5 FC criteria, for simplicity. When using the FC setting, implemented in the Excel addin, the researcher selects a proportion of the all genes on the chip, and this selection is also utilised for the permutated control data set, however the settings from the full data set SAM analysis are maintained (s_0, and pi_0). The FC hurdle setting can therefore change the resulting q-values as genes that pass a certain FC when the original sample categories are used, are less likely to generate high d(i) when the categories are permutated. To test the extent of this effect, we performed SAM at several different FC hurdles within Excel and scored the number of genes reported at the final 1.5 FC criteria. A difference in the number of genes reported as significant above the final FC would indicate that by using the Excel SAM FC restriction setting, during SAM, we change the outcome by altering the d(i) distribution of the permutation data set.

**Figure 1:**
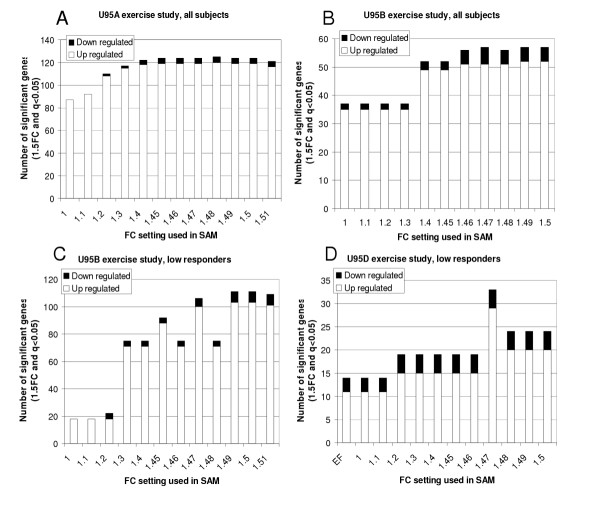
**FC effects on the endurance training data set: **SAM analysis was used at various fold changes studying the exercise data set while scoring genes with a q-value of <0.05 and FC>1.5. This was done to asses the effect of the fold change option in the SAM Excel addin on genes reported as significant at a higher fold change. The figure shows the number of scored genes using 4 different chip and sample combinations: **(A) **All eight subjects before and after training (totally 16 arrays) were compared in a paired analysis using the U95A chips. **(B) **All eight subjects before and after training (totally 16 arrays) were compared in a paired analysis using the U95B chips. **(C) **The reduced group consisting of low four low responders (totally 8 arrays) were compared in a paired analysis using the U95B chips. **(D) **The reduced group consisting of low four low responders (totally 8 arrays) were compared in a paired analysis using the U95D chips.

We used three different biological data sets to assess how wide-spread any effects were. The first data set was a paired data set from a human skeletal muscle study which examined subjects before and after endurance training using the U95A-E platform (Affymetrix) [3]. The data was RMA normalized [4-6] and SAM was performed using different biological sample subgroups (groups were formed on the basis of a variety of physiological parameters) and chipset identity (A, B or D). Changing the FC setting in SAM (Excel) altered the final list of significant genes at the 1.5 FC criteria (Figure 1A–D). Importantly, the effect was not uniform across all conditions. For some samples a sequential increase in the FC hurdle in SAM correlated with an increased number of reported genes (Figure 1A–B) while under other conditions a very small change in FC had an apparently random impact on the composition of the significant gene list (e.g. Figure 1C–D). (The number of genes that passed each fold change criteria can be found in [Additional file 1]).

**Figure 2:**
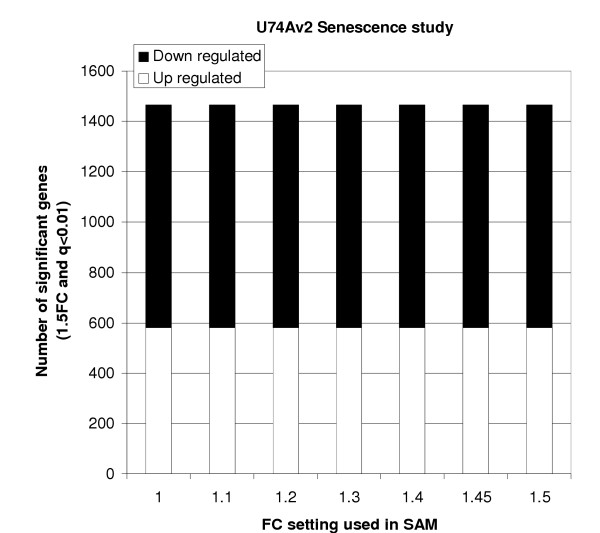
**FC effects on the senescence data set: **SAM analysis was used at various fold changes using the senescence data set while scoring genes with a q-value of <0.01 and FC>1.5. A comparison between non-senescent cells and senescent cells was used (two replicates of the senescent cells and four replicates of the non-senescent cells).

The second data set was derived from an *in-vitro *mouse senescence study performed on U74Av2 chips [7] and normalized using RMA [4-6]. When comparing two of the time points during the induction of senescence we were unable to observe any effect of FC selection within SAM on the yield of significant genes (Figure 2). We believe this reflected the very low q-values obtained when originally using SAM, which in turn most likely reflects the low experimental variation that one can achieve using *in-vitro *models.

**Figure 3:**
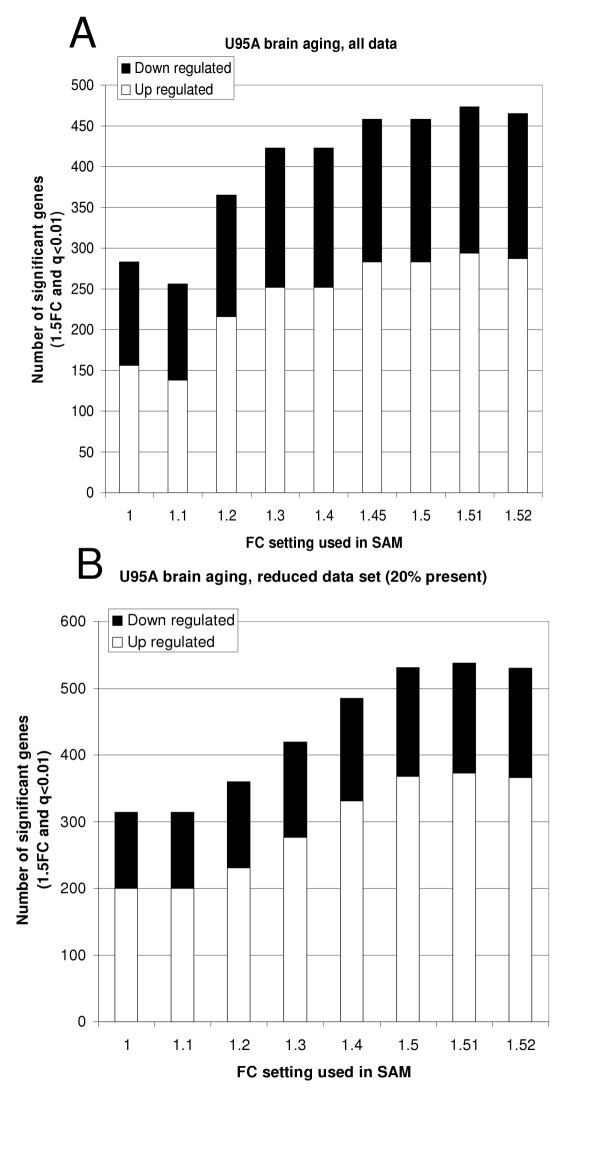
**FC effects on the brain aging data set: **SAM analysis at various fold changes using the brain aging data set (11 old samples and 10 young samples) while scoring genes with a q-value of <0.01 and FC>1.5. We used both the full data set and the reduced data set suggested by the authors. **(A) **The full data set. **(B) **The reduced data set (20% "present" calls by D-chip").

The third data set is derived from a study of the aging human brain, containing 21 samples split into two categories, young and old (unpaired data) [8], and normalised using D-chip [1]. When varying the FC hurdle within SAM we again saw a large impact on the number of genes reported as being significant, above the 1.5 FC criteria (a q-value threshold of 1% was considered statistically significant similarly to the original report) (Figure 3A–B). The 'significant' gene list increased from 283 genes when using no FC hurdle (during SAM in Excel) to a maximum of 465 significant genes (67% more) when using a ***1.51 ***FC hurdle within Excel SAM (Figure 3A).

A similar effect was observed when we examined the methods utilised by the original authors (20% "Present" call by D-chip). No FC hurdle generates 314 significant genes and a 1.51 FC generates 538 genes (Figure 3B). This is a 71% increase in "significant" genes associated with aging in human brain, despite the fact that the FC hurdle utilised in SAM was actually greater than the final FC of 1.5! Similar effects arise when other final FC criterions are used during SAM. For example if we only consider the significant genes that pass a final FC >2 criteria in the aging brain study, then 5% more >2 FC genes can be found if the FC hurdle within SAM is set to 2.0 compared to 1.5. Clearly, genes that are modulated to a large extent may be of high biological interest and thus our observation is important. Also, there are 73 more significant genes at the FC hurdle of 1.51 if the reduced data set is used compared the full data set (less genes in the data set gave more significant genes). This indicates that Present/Absent filtering also influences the outcome of the analysis, in this case increasing the number of genes defined as being modulated.

### Changing the FC setting in SAM changes the reported q-value

**Figure 4:**
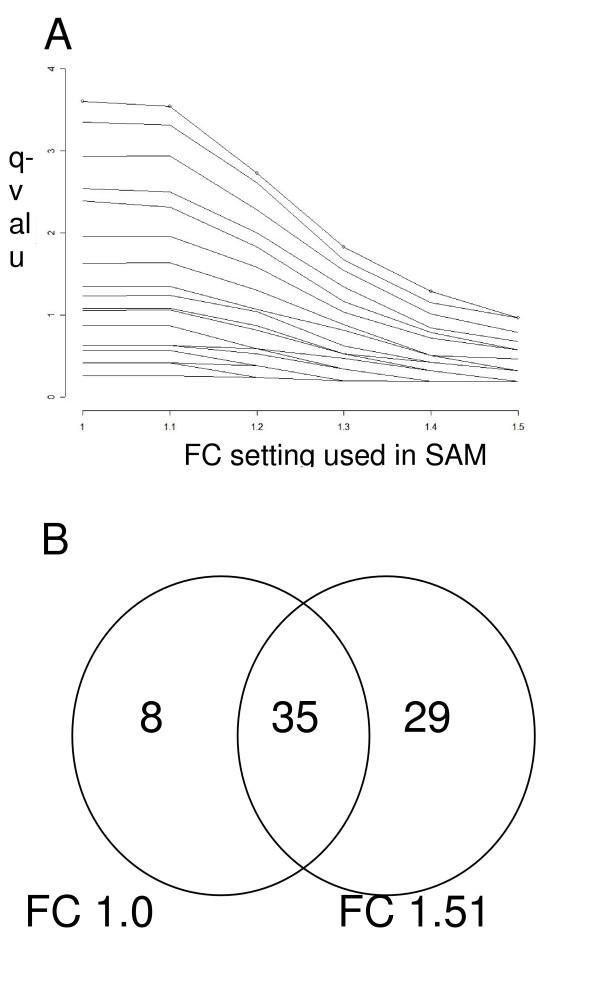
**FC effects individual q-values: **q-values of all genes scored as significant in the brain aging study (reduced data set) at FC 1.51 at other FC settings. Genes only acquires discrete q-values and all 538 genes are shown, but overlap. **(B) Running SAM with different FC settings changes the biological interpretation: **Venn diagram comparing the number of significantly overrepresented classifications (EASE score <0.05) using the reduced brain aging data set analysed either with a 1.0 FC setting (314 genes) or a 1.5 FC setting (538 genes).

The analysis presented above demonstrates that the q-value obtained for a specific gene depends on the FC hurdle applied during SAM in Excel. To monitor the q-values generated, for individual genes, we obtained the q-values during all SAM calculation using various FC hurdles for all genes that were reported as significant at the 1.51 FC setting (538 genes) in the brain aging study (Figure 4A). The highest q-value for a subset of the genes that passed the final fold change criteria was 3.6% when SAM was performed with a 1.0 FC hurdle. The same genes appear as being significant when the "optimal" (in the sense that these setting produced the largest significant gene list) Excel SAM FC hurdle setting 1.51 is used while the highest q-value reported was now only 0.97%.

### Running SAM using different FC settings can change data interpretation

Often the main reason for carrying out a microarray experiment is to gain greater insight into the molecular processes that contribute to a complex biological phenotype. One standard method for carrying out such analysis is the use of gene-grouping, such as Gene ontology classification [9]. To address whether the biological interpretation may be influenced, we compared overrepresentation of classifications using EASE [10]. We selected the 314 genes and the 538 genes obtained in the brain aging study using the 1.0 and a 1.51 FC hurdles (described above) and calculated the overrepresented classifications. There was a considerable difference in the number of significantly over-represented classifications identified by EASE (figure 4B). 35 classifications were overrepresented in both gene lists, 8 were found in the GO analysis of the 1.0 FC list and 29 were unique to the 1.51 FC list. We feel it is unlikely that greater inclusion of randomly determined genes (i.e. false positives) would give rise to a significant increase in statistically enriched functional groups. This indicates that both the number of genes and the interpretation may be substantially altered by arbitrary data filtering, as exemplified by the use of the FC hurdle during the operation of Excel SAM or through the application of present-absent calls using e.g. D-chip.

## Conclusion

The average researcher is highly dependent on the use of 'standard procedures' for their microarray analysis. An arbitrary filtering option in the Microsoft Excel Addin (we have called this the FC hurdle to distinguish from FC criteria – which is the *final *FC value selected by the researcher, to define a functionally significant change in gene expression) or data exclusion (e.g. present or absent call thresholds) can impact on a study, in a less than predictable manner. A clear problem also arises when SAM is utilised on different software platforms. In the R package *Siggenes*, no FC hurdle criteria can be made unless an additional programming function is implemented by the individual researcher. This is in contrast to the more widely used Microsoft Excel *SAM Addin *where the researcher can introduce a FC hurdle prior to the q-value calculations (while the SAM algorithm maintains the initial parameters from the analysis of the full data set). It is often unclear in the literature if SAM was performed in Excel or R; and if an FC hurdle was applied within SAM or if the fold change criteria was introduced after completing the operation of SAM, in EXCEL.

Indeed, one might question how robust the SAM methodology is, if it is heavily dependent on both pre SAM data selection and within SAM (Excel) data filtering. However, one of the appreciated strengths of SAM is that the real data set is used to estimate experimental variation. One could also question whether it is valid to reduce the data set prior to using SAM. It would seem intuitive that much of the data being removed using a FC hurdle during SAM operation would be below an acceptable response level to be considered as being biologically relevant. However, the FC hurdle may also remove data that is essential for an accurate estimation of the experimental variation. The challenge would then be to remove 'genuine' noise from non-expressing genes without removing genes that are genuinely expressed and necessary to approximate the data set variation. The effects will be pronounced in data sets demonstrating a large range of gene expression FC's or where significant inter experimental variation exists. It is clear, that investigators must be made aware that the impact of 'qualification criteria for inclusion' and 'out-put filtering' is less than predictable, when using SAM.

## Methods

### Data sets

Three data sets were used. The first is a human skeletal muscle study, comparing eight subjects across the U95A-E Affymetrix chips before and after endurance training [3]. A subset of this data set can be derived by creating a low responder group based on their improvement upon training. This subset is referred to as the low responder group in figure 1. The data can be obtained from the authors (JAT). The second data set is a part of an mouse in-vitro senescence study performed on U74Av2 (Affymetrix) [7]. Two conditions were selected for comparison: EpiA1-ts58 cells at the basal condition and EpiA1-ts58 cells after 72 hours of senescence induction. This data set can be downloaded from the journal web site in a MAS5 (Affymetrix microarray suite 5) normalised format or will be distributed in RMA normalised format by the authors (OL). The third data set is a brain aging study performed on the U95A chip (Affymetrix) and normalised using D-chip [8]. The authors separated their data set into two groups: young (<43 years old) and old (>72 years old) and we used the same grouping in our analysis. The authors further used a data discrimination based on "present" calls given by D-chip (20% present in all samples). We used both the full data set and the 20% data set which is referred to as the reduced data set. The data set can be access at GEO, accession number GSE1572.

### SAM analysis

SAM analysis was performed using the Microsoft Excel addin v1.21. The analysis was performed using different settings of the "Fold change". We also performed SAM analysis using the R (R-project.org) package "siggenes" as a comparison to the Excel addin without using the fold change setting with consistent results.

### Classification analysis

To look for overrepresented classifications we used EASE [10]. We used all possible classifications and considered a classification as positively overrepresented if the EASE score was lower then 0.05.

## Abbreviations

SAM – significance analysis of microarrays

FDR – false discovery rate

FC – fold change

EF – extraction factor

MAS5 – microarray suite 5

RMA – robust multi array average

GEO – gene expression omnibus

## Authors' contributions

OL – Hypothesis generation, practical work with all data sets and drafting of the manuscript

CW – Study design and revising the manuscript.

JAT – Hypothesis generation, practical work on the human muscle data section and drafting of the manuscript

## Supplementary Material

Additional File 1 Genes that pass various FCs The additional file describes how many genes that passed various FCs from all three data sets Click here for file

## References

[B1] Li C, Hung Wong W (2001). Model-based analysis of oligonucleotide arrays: model validation, design issues and standard error application. Genome Biol.

[B2] Tusher VG, Tibshirani R, Chu G (2001). Significance analysis of microarrays applied to the ionizing radiation response. Proc Natl Acad Sci U S A.

[B3] Timmons JA, Larsson O, Jansson E, Fischer H, Gustafsson T, Greenhaff PL, Ridden J, Rachman J, Peyrard-Janvid M, Wahlestedt C, Sundberg CJ (2005). Human muscle gene expression responses to endurance training provide a novel perspective on Duchenne muscular dystrophy. Faseb J.

[B4] Irizarry RA, Bolstad BM, Collin F, Cope LM, Hobbs B, Speed TP (2003). Summaries of Affymetrix GeneChip probe level data. Nucleic Acids Res.

[B5] Bolstad BM, Irizarry RA, Astrand M, Speed TP (2003). A comparison of normalization methods for high density oligonucleotide array data based on variance and bias. Bioinformatics.

[B6] Irizarry RA, Hobbs B, Collin F, Beazer-Barclay YD, Antonellis KJ, Scherf U, Speed TP (2003). Exploration, normalization, and summaries of high density oligonucleotide array probe level data. Biostatistics.

[B7] Larsson O, Scheele C, Liang Z, Moll J, Karlsson C, Wahlestedt C (2004). Kinetics of senescence-associated changes of gene expression in an epithelial, temperature-sensitive SV40 large T antigen model. Cancer Res.

[B8] Lu T, Pan Y, Kao SY, Li C, Kohane I, Chan J, Yankner BA (2004). Gene regulation and DNA damage in the ageing human brain. Nature.

[B9] Harris MA, Clark J, Ireland A, Lomax J, Ashburner M, Foulger R, Eilbeck K, Lewis S, Marshall B, Mungall C, Richter J, Rubin GM, Blake JA, Bult C, Dolan M, Drabkin H, Eppig JT, Hill DP, Ni L, Ringwald M, Balakrishnan R, Cherry JM, Christie KR, Costanzo MC, Dwight SS, Engel S, Fisk DG, Hirschman JE, Hong EL, Nash RS, Sethuraman A, Theesfeld CL, Botstein D, Dolinski K, Feierbach B, Berardini T, Mundodi S, Rhee SY, Apweiler R, Barrell D, Camon E, Dimmer E, Lee V, Chisholm R, Gaudet P, Kibbe W, Kishore R, Schwarz EM, Sternberg P, Gwinn M, Hannick L, Wortman J, Berriman M, Wood V, de la Cruz N, Tonellato P, Jaiswal P, Seigfried T, White R (2004). The Gene Ontology (GO) database and informatics resource. Nucleic Acids Res.

[B10] Hosack DA, Dennis GJ, Sherman BT, Lane HC, Lempicki RA (2003). Identifying biological themes within lists of genes with EASE. Genome Biol.

